# Dieckol, Derived from the Edible Brown Algae *Ecklonia cava,* Attenuates Methylglyoxal-Associated Diabetic Nephropathy by Suppressing AGE–RAGE Interaction

**DOI:** 10.3390/antiox12030593

**Published:** 2023-02-27

**Authors:** Chi-Heung Cho, Guijae Yoo, Mingyeong Kim, Ulfah Dwi Kurniawati, In-Wook Choi, Sang-Hoon Lee

**Affiliations:** 1 Division of Functional Food Research, Korea Food Research Institute, Wanju 55365, Republic of Korea; 2 Department of Food Biotechnology, University of Science and Technology, Daejeon 34113, Republic of Korea

**Keywords:** dieckol, receptor for advanced glycation end product, molecular docking, methylglyoxal, diabetic nephropathy, brown seaweed, Nrf2/Glo-1/AGE signaling pathway

## Abstract

The formation of advanced glycation end products (AGE) is linked to the pathogenesis of diabetic nephropathy. The aim of this work was to assess the therapeutic potential and underlying mechanism of action of dieckol (DK), isolated from *Ecklonia cava*, on renal damage induced by methylglyoxal (MGO) in mouse glomerular mesangial cells. The antiglycation properties of DK were evaluated using ELISA. We conducted molecular docking, immunofluorescence analysis, and Western blotting to confirm the mechanism by which DK prevents AGE-related diabetic nephropathy. DK treatment exhibited antiglycation properties through the inhibition of AGE production, inhibition of cross-linking between AGE and collagen, and breaking of its cross-linking. DK pretreatment exhibited protective effects on renal cells by suppressing MGO-induced intracellular reactive oxygen species (ROS) formation, intracellular MGO and AGE accumulation, activation of the apoptosis cascade and apoptosis-related protein expression, activation of receptor for AGE (RAGE) protein expression, and suppression of the glyoxalase system. Furthermore, DK exhibited a stronger binding affinity for RAGE than AGE, which was confirmed as exerting a competitive inhibitory effect on the AGE–RAGE interaction. These results demonstrated that DK is a potential natural AGE inhibitor that can be utilized to prevent and treat AGE-induced diabetic nephropathy.

## 1. Introduction

Diabetes mellitus (DM) is a chronic metabolic disorder that causes long-term damage to various organs by promoting secondary complications. Although diabetes is exacerbated by various factors, abnormally elevated synthesis and accumulation of advanced glycation end products (AGEs) in patients with chronic hyperglycemia is considered a trigger in the pathogenesis of chronic diabetic complications. AGEs are complex heterogeneous compounds that are naturally synthesized during the glycation reaction through several phases [1]. Increased accumulation of AGE in chronic hyperglycemic conditions leads to diverse diabetic complications, including retinopathy, neuropathy, and diabetic nephropathy. Diabetic nephropathy occurs in approximately 35–45% of patients with diabetes [2]. Particularly, intracellular glycation reaction, cross-linking with collagen, and AGE–RAGE (receptor for AGEs) interactions are known to be key mechanisms leading to AGE-related diabetic nephropathy. Therefore, AGEs are considered key biomarkers in patients with chronic hyperglycemia because they are responsible for the pathogenesis of diabetic complications [3,4]. Consequently, inhibiting AGE formation or aberrant accumulation, suppressing AGE–RAGE interaction, and regulating RAGE protein expression have been proposed as effective strategies for preventing or delaying AGE-induced diabetic complications.

Methylglyoxal (MGO), an α-dicarbonyl compound, is naturally produced by various metabolic pathways, including glycolysis. Moreover, MGO-derived AGE directly reacts with lysine and arginine residues in proteins, resulting in protein denaturation and dysfunction [5]. Furthermore, dicarbonyl stress by MGO increases oxidative damage to proteins, intracellular ROS generation, and renal cell apoptosis [6,7]. In addition, increased production of MGO and MGO-protein adducts is associated with the pathogenesis of diabetic nephropathy. Particularly, MGO accumulates in the kidneys and causes renal dysfunction, such as decreased glomerular filtration rate due to hypertrophy of the glomerular basement membrane [2,8]. MGO is converted to nontoxic D-lactate by the detoxification of glyoxalase-1 (Glo-1) in the kidney and is excreted. Since nuclear factor erythroid-2-related factor 2 (Nrf2) is involved in regulating the mRNA, protein, and activity of glo-1, Glo-1 enzyme activity is closely related to Nrf2 expression. Nrf2 is a transcription factor that mediates the antioxidant response element (ARE)-dependent activation of antioxidant enzymes, which play a critical role in protecting cells from oxidative stress-induced damage [2]. Therefore, upregulating Nrf2 not only promotes MGO detoxification through glo-1 activation, but also increases the expression of antioxidant enzymes, which can help suppress MGO-induced kidney dysfunction.

*Ecklonia cava* (*E. cava*) is an edible brown alga found along the Pacific coast around Jeju Island in Korea and is used as a food, cosmetic, and medicinal ingredient. *E. cava* is rich in bioactive components with various biological activities, such as fucoidans, phlorotannins, minerals, polysaccharides, dietary fiber, peptides, and carotenoids, and is used as a major ingredient in industrial applications in pharmaceuticals, cosmetics, and functional foods. Among the bioactive components found in *E. cava*, phlorotannin with a phloroglucinol structure has been demonstrated to exhibit diverse physiological activities such as anti-inflammatory, whitening, anticancer, antioxidant, and antibacterial properties [9,10]. Furthermore, *E. cava* and *E. cava*-derived phlorotannins have been shown to have antidiabetic activities by reducing human umbilical vein endothelial cell damage by hyperglycemia-induced glucotoxicity, activating both the AMPK/ACC and PI3K/Akt signaling pathways and inhibiting α-glucosidase and α-amylase activity, both in vitro and in vivo [2,9].

In this study, the therapeutic potential of dieckol isolated from *E. cava* on AGE-induced renal damage was evaluated through its antiglycation properties, AGE–RAGE interaction inhibitory ability, RAGE protein regulating ability, and antiapoptotic properties in mouse glomerular stromal cells.

## 2. Materials and Methods

### 2.1. Chemicals

Dieckol (DK) isolated from *Ecklonia cava* were kindly provided by Prof. You-Jin Jeon (Jeju Notional University, Republic of Korea) [11]. MGO and aminoguanidine (AG) were ordered from Sigma-Aldrich (St. Louis, MO, USA). Reagents used in all experiments were purchased in analytical grade. Information on the antibody used for Western blot is presented in Supplementary File (Table S1).

### 2.2. Antiglycation Property

To evaluate the antiglycation effect of DK, the inhibitory effect on AGE formation was evaluated by partially modifying the method of Kiho et al. [12]. Bovine serum albumin (10 mg/mL), glucose (2 M), and fructose (2 M) were dissolved in 50 mM phosphate-buffered saline (PBS, pH 7.4) containing 0.02% (*w/w*) sodium azide and mixed with DK (1, 5, and 20 μM). Mixture was reacted at 37 °C for seven days. As a positive control, 50 mM PBS was added instead of DK, and aminoguanidine (AG, 0.5 mM) was used. AGE formation was measured using a fluorescence microplate reader (Molecular Devices, Sunnyvale, CA, USA) at 350 nm (excitation)/450 nm (emission).

To further evaluate the antiglycation ability of DK, the cross-link generation inhibitory effect between AGE and collagen was measured by slightly modifying the method of Do et al. [13]. Horseradish peroxidase-labeled (HRP) AGE (5 μg/mL) was mixed with DK (1, 5, and 20 μM), transferred to a collagen-coated 96-well plate, and cross-links formed between AGE and collagen at 37 °C for 18 h. AG (0.5 mM) was used as a positive control. Then, TMB substrate solution was added the reaction for 3 min.

To evaluate the breaking ability of DK on the cross-links formed between AGE and collagen, HRP-labeled AGE was reacted in a collagen-coated 96-well plate at 37 °C for 4 h to form a cross-link. Then, DK was added and reacted at 37 °C for 18 h. Alagebrium (ALT-711, 0.5 mg/mL), a cross-link breaker, was used as a positive control. After incubation, TMB substrate solution was added, the reaction was allowed to proceed for 3 min. The inhibiting and breaking abilities were measured using a microplate reader (Molecular Devices, Sunnyvale, CA, USA) at 450 nm.

### 2.3. Cell Culture

The mouse glomerular mesangial cells lines were obtained from the American Type Culture Collection. The mesangial cells were cultured DMEM/F12 medium containing 5% (*v/v*) fetal bovine serum, 4-(2-hydroxyethyl)-1-piperazineethanesulfonic acid (HEPES; 14 mM), penicillin (100 U/mL), and streptomycin (100 μg/mL) and was cultured under conditions of 5% CO_2_, 95% humidity, and 37 °C.

### 2.4. Cell Viability

The ability of DK to protect renal cells from MGO-induced oxidative stress was evaluated by MTT assay. Mesangial cells were seeded (3 × 10^4^/well) in a 96-well plate and the plates were incubated under culture conditions (37 °C for 6 h). After treatment with DK (1, 5, and 20 μM) and incubation for 1 h, MGO (1 mM) was added and incubated at 37 °C for 23 h. AG (0.5 mM) was used as a positive control. After removing the supernatant, the MTT reagent was allowed to react for 3 h to form formazan The resulting formazan products from cells were dissolved with the addition of DMSO. The amount of MTT formazan was determined by measuring absorbance using a microplate reader (Infinite M200; Tecan Austria GmbH, Grödig, Austria) at 570 nm.

### 2.5. Intracellular ROS Production

The effect of DK on intracellular reactive oxygen species (ROS) production by MGO was performed by DCFH-DA assay with a modification of the method of Cho et al. [14]. Mesangial cells were seeded (3 × 10^4^/well) in a 96-well plate and the plates incubated under culture conditions (at 37 °C for 6 h). After treatment with DK (1, 5, and 20 μM) and incubation for 1 h, MGO (1 mM) was added and incubated at 37 °C for 23 h. AG (0.5 mM) was used as a positive control. After removing the supernatant, the DCFH-DA reagent was allowed to react for 30 min. The intracellular ROS production was determined using a fluorescence microplate reader (Infinite M200; Tecan) at an excitation and emission wavelength of 485 and 530 nm, respectively.

### 2.6. In Silico Molecular Docking Study

Molecular docking studies were performed according to the CDOCKER protocol in Discovery Studio Software (BIOVIA Corp., CA, USA). CDOCKER is a molecular dynamics model based on the Chemistry at Harvard Macromolecular Mechanics (CHARMm) algorithm. The high-resolution crystal structure of RAGE (PDB code: 2MOV) was retrieved from the Protein Data Bank PDB (http://www.rcsb.org/pdb, access date: 16 April 2021). The RAGE was prepared by the “Clean protein” module in software. Incomplete amino acid residues were supplemented, hydrogens were added to the protein, and energy minimization was performed using the CHARMm force field. The RAGE substrate ligand methylglyoxal-derived hydroimidazolone-1 (MG-H1), AGE inhibitor AG (positive control), and DK were docked in a flexible manner to achieve a more realistic view of the possible protein–ligand interactions. The result was obtained from the calculated –CDOCKER energy (protein–ligand interaction, and the ligand strain energy). The score of the substrate ligand is one of the criteria for choosing a docked pose, with higher values indicating more favorable binding.

### 2.7. Intracellular MGO Concentration

To evaluate the inhibitory effect of DK on MGO accumulation in renal cells, ELISA was performed using Methylglyoxal Competitive kit (OxiSelect™; Cell Biolabs Inc. San Diego, CA, USA). Mesangial cells were seeded (1.0 × 10^6^/well) in a 6-well plate and cultured for 6 h. To induce the intracellular MGO accumulation, MGO (1 mM) was added for 23 h after treatment with DK (1, 5, and 20 μM) for 1 h. AG (0.5 mM) was used as the positive control. The supernatant was removed, the cells were collected using trypsin-EDTA, and cell lysis was performed using PRO-PREP™ Protein Extraction Solution (iNtRON Biotechnology, Seongnam, Korea). The experiment was conducted as per the kit’s manual.

### 2.8. Intracellular AGEs Accumulation

To evaluate the inhibitory effect of DK on AGE accumulation in renal cells, AGE antibody/DAPI double-immunofluorescence analysis was performed. Mesangial cells were seeded (2.0 × 10^5^/well) in a chamber slide and cultured for 6 h. To induce the intracellular accumulation of AGE, MGO (1 mM) was treated for 23 h after treatment with DK (1, 5, and 20 μM). AG (0.5 mM) was used as the positive control. After removing the supernatant, the cells were fixed using 4% (*v/v*) formalin solution at 23 °C for 15 min. Then, 0.1% (*v/v*) Triton X-100 was treated for 5 min. Blocking was performed using 1% (*v/v*) BSA for 30 min. Thereafter, the cells were treated with AGE antibody and incubated at 4 °C for 24 h, followed by Alexa 288 secondary antibody at 23 °C for 2 h. Finally, after treating the cells with a mounting solution containing DAPI, AGE in renal cells was observed using a fluorescence microscope (Zeiss Axio Observer A1, ZEISS, Jena, Germany).

### 2.9. Apoptosis Analysis

The inhibitory effect of DK on MGO-induced renal cell apoptosis was evaluated using a MUSE flow cytometry system (Merck Millipore, Sydney, Australia). Mesangial cells were seeded (1.0 × 10^6^/well) in a 6-well plate and cultured for 6 h. To induce cell apoptosis, MGO (1 mM) was treated for 23 h after treatment with DK (1, 5, and 20 μM). AG (0.5 mM) was used as the positive control. The supernatant was removed, and cells were collected using trypsin-EDTA and Muse™ Annexin V & Dead Cell reagent (Luminex, TX, USA). The inhibitory effect of DK on apoptosis induced by MGO-induced caspase 3/7 activation was evaluated using the MUSE caspase 3/7 assay kit. Total apoptotic cells (early and late apoptotic cells) and caspase 3/7 activation were analyzed using a MUSE cell analyzer.

To visually confirm the inhibitory effect of DK on MGO-induced renal cell apoptosis, we used Hoechst 33342/PI double staining. Mesangial cells were seeded (2.0 × 10^5^/well) in chamber slides and cultured for 6 h. To induce cell apoptosis, MGO (1 mM) was treated for 23 h after treatment with DK (1, 5, and 20 μM). AG (0.5 mM) was used as the positive control. After removed the supernatant, the cells were fixed in 4% (*v/v*) formalin solution at 23 °C for 10 min. The cells were treated with 0.2% (*v/v*) Triton X-100 for 10 min. Finally, the cells were stained with Hoechst 33342 (2 μg/mL) and PI (10 μg/mL) for 30 min. Renal cell apoptosis was observed using a fluorescence microscope (Zeiss Axio Observer A1, ZEISS, Jena, Germany).

### 2.10. Western Blot

The pretreated cells were scraped and proteins were extracted using PRO-PREP containing 1% (*v/v*) protease and phosphatase. Protein samples were adjusted to the same amount by protein quantification and loaded onto gel (Any kD Mini-PROTEAN TGX Stain-Free Gel; Bio-Rad, Hercules, CA, USA); electrophoresis was performed at 200 volts. Proteins were then transferred to polyvinylidene difluoride membranes using the Transfer Kit (Trans-Blot Turbo RTA Mini 0.2 μm Nitrocellulose; Bio-Rad). After blocking the polyvinylidene difluoride membrane with 5% (*w/v*) skim milk at 23 °C for 2 h, it reacted with primary antibodies at 4 °C for 24 h. Information on the antibody used for Western blot is presented in Supplementary File (Table S1). After sufficient washing of the membrane, it was reacted with a secondary antibody (HRP-labeled goat anti-rabbit IgG or goat anti-mouse IgG) at 23 °C for 2 h. Protein bands were measured using the ChemiDoc XRS + imaging system (Bio-Rad, CA, USA), and quantification of the measured proteins was performed using Image Lab software.

### 2.11. Statistical Analysis

All experiments were performed independently at least three times. The calculated results were expressed as mean ± standard deviation values. GraphPad Prism version 9.0 (GraphPad Software, Inc., San Diego, CA, USA) was used to statistically analyze and draw graphs. One-way analyses of variance followed by Tukey’s honestly significant difference test (*p* < 0.05) were applied to determine the significance of the differences among the means.

## 3. Results

### 3.1. Antiglycation Property of DK

**Figure 1:**
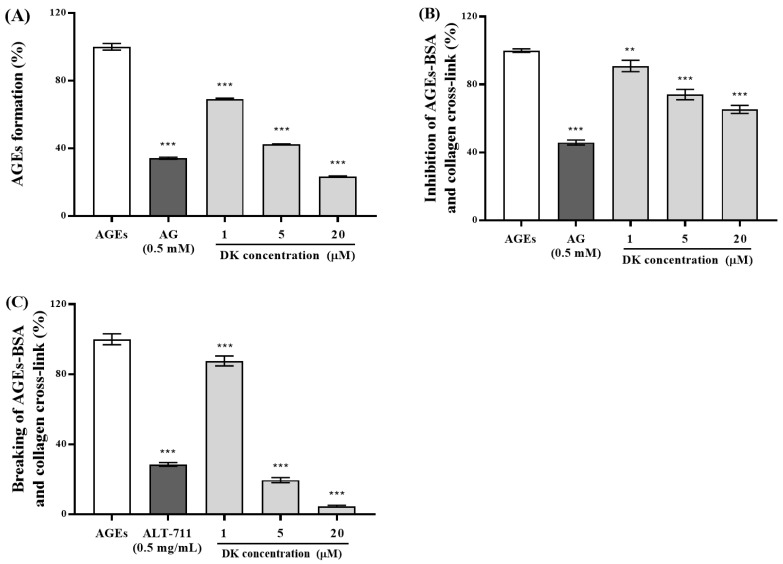
The inhibitory ability of dieckol (DK) isolated from *Ecklonia cava* (*E. cava*) on an in vitro glycation reaction: (**A**) evaluation of DK’s ability to inhibit AGE formation; (**B**) evaluation of DK’s ability to inhibit cross-linking formation between AGE and collagen; (**C**) evaluation of DK’s ability to break cross-links formed between AGE and collagen. All experiments were independently repeated three times. Data are presented as mean ± standard deviation (*n* = 3) (** *p* < 0.01 and *** *p* < 0.001 vs. AGE).

In this study, we evaluated the in vitro antiglycation properties of DK using a fluorescence-based AGE formation inhibitory assay. As shown in Figure 1A, incubation with DK (1, 5, and 20 μM) or AG (0.5 mM) significantly (*** *p* < 0.001) decreased AGE formation to 69.15 ± 0.47%, 42.49 ± 0.16%, 23.35 ± 0.36%, and 34.14 ± 0.58%, respectively, compared to the nontreated group.

To investigate the formation of AGE-collagen cross-link inhibitory properties of DK, we used ELISA. Treatment with DK (1, 5, and 20 μM) dose-dependently suppressed cross-linking (Figure 1B). Moreover, cotreatment with DK (20 μM) or AG (0.5 mM) significantly (*** *p* < 0.001) suppressed AGE-collagen cross-linking formation to 65.34 ± 2.35% and 45.83 ± 1.44%, respectively.

We used ELISA to determine the strength of cross-link breaking to investigate AGE-collagen cross-link-breaking ability of DK. As shown in Figure 1C, treatment with AGE-BSA markedly increased AGE-collagen cross-linking, while treatment with DK at concentrations of 1, 5, and 20 μM dramatically decreased AGE-collagen cross-links to 87.57 ± 2.84%, 19.52 ± 1.45%, and 4.62 ± 0.51%, respectively. In particular, 20 μM DK treatment exhibited a more effective breaking ability than ALT-711, a representative cross-link breaker used as a positive control.

### 3.2. Protective Effect of DK against MGO-Induced Renal Damage

**Figure 2:**
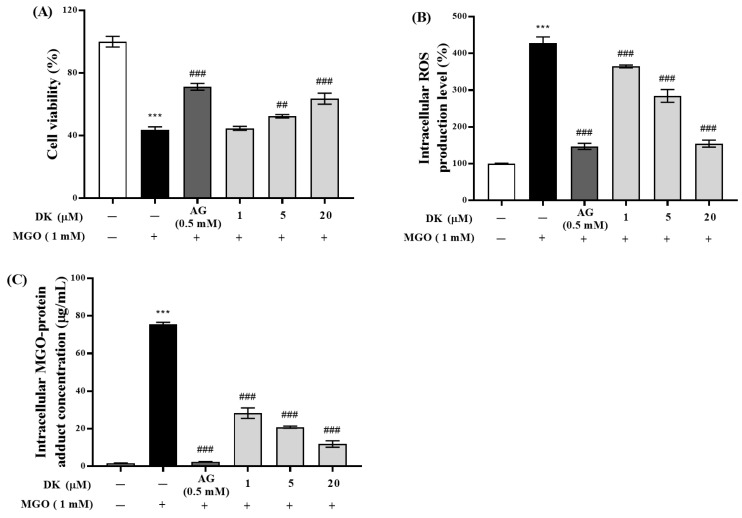
Preventive effect of DK against MGO-induced renal cell damage on mouse glomerular mesangial cells. It was confirmed through MTT assay that DK had no cytotoxicity up to a concentration of 20 μM on mouse glomerular mesangial cells: (**A**) Renal cell protective effect of DK against MGO-induced oxidative stress induced was confirmed by MTT assay; (**B**) DCFH-DA assay was performed to evaluate the intracellular antioxidant activity of DK against intracellular oxidative stress induced by MGO treatment; (**C**) By measuring the intracellular MGO-protein adduct concentration, the inhibitory efficacy of DK on intracellular MGO accumulation was evaluated. All data are expressed as mean ± standard deviation (*n* = 3) (*** *p* < 0.001 vs. nontreated cells and ## *p* < 0.01, ### *p* < 0.001 vs. MGO-treated cells).

The viability of mouse glomerular mesangial cells was not significantly different from that of the control group after treatment with DK (1, 5, and 20 μM) for 24 h, indicating that DK was not toxic to mesangial cells under treatment conditions. Exposing mesangial cells to MGO induced an approximately 50% decrease in cell viability (Figure 2A). However, pretreatment with DK exhibited a concentration-dependent effect on protecting mesangial cells from MGO-induced dicarbonyl stress, and 20 μM DK increased the viability to 63.6% (### *p* < 0.001).

DCFH-DA is oxidized to the fluorescent compound DCF by oxidative stress in cells, which indirectly reflects the ROS levels in mesangial cells. MGO treatment induced overproduction of intracellular ROS to 427.43 ± 17.29% in mesangial cells compared to the normal group (*** *p* < 0.001), while pretreatment with DK at concentrations of 1, 5, and 20 μM significantly suppressed MGO-induced intracellular ROS generation to 364.42 ± 3.80%, 284.27 ± 17.39%, and 154.63 ± 9.88% (### *p* < 0.001), compared to the normal group (Figure 2B).

Treatment with 1 mM MGO increased the intracellular MGO cross-linked protein concentrations up to 75.55 ± 1.03 μg/mL in mesangial cells (*** *p* < 0.001) (Figure 2C). However, pretreatment with DK at concentrations of 1, 5, and 20 μM significantly decreased MGO-induced intracellular MGO accumulation to 28.28 ± 0.04, 20.84 ± 2.79, and 11.86 ± 1.74 μg/mL (### *p* < 0.001) in mesangial cells. AG (0.5 mM), used as the positive control, reduced the intracellular MGO concentration to 2.46 ± 0.04 μg/mL.

### 3.3. Molecular Docking Analtsis and Effect of DK on AGE/RAGE Axis in Renal Cells 

**Figure 3:**
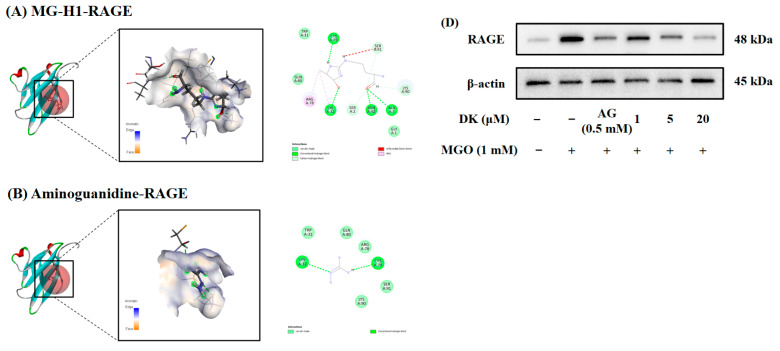

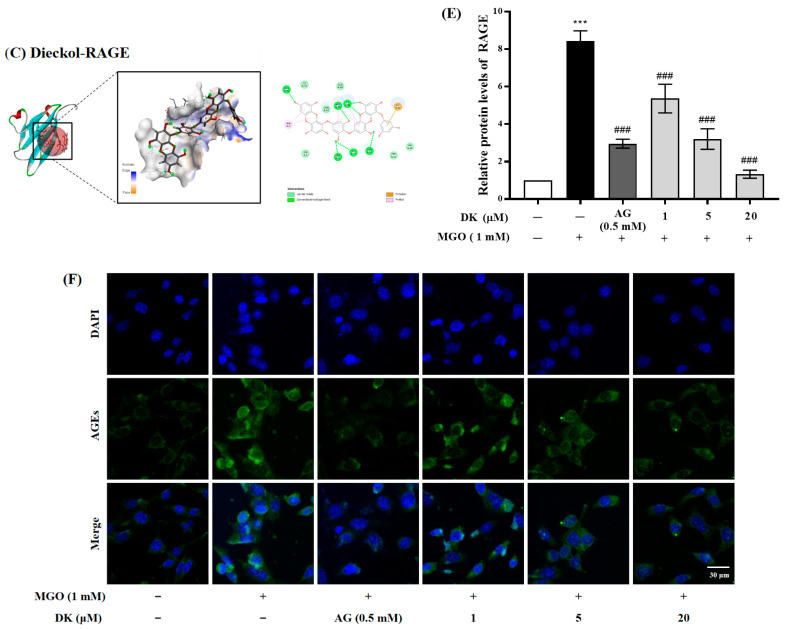
The crystal structures and 2D interactions of the docked ligands at the active site of RAGE (PDB code: 2MOV): (**A**) MGO-H1-RAGE; (**B**) aminoguanidine-RAGE; (**C**) dieckol-RAGE; (**D**) Inhibitory effect of DK on RAGE protein expression; (**E**) Relative RAGE protein band intensity; β-actin was used as an internal control; (**F**) Intracellular AGE accumulation inhibitory ability of DK. Representative images of AGE antibody/DAPI double staining. All data are expressed as mean ± standard deviation (*n* = 3) (*** *p* < 0.001 vs. nontreated cells and ### *p* < 0.001 vs. MGO-treated cells).

RAGE is an AGE specific receptor that binds to AGEs and MGO, ultimately contributing to long-term oxidative damage and promoting diabetic nephropathy. Molecular docking estimates the ligand-receptor binding energy by exploring the 3D structure of the ligand employed within the active site of the receptor and evaluating key phenomena involved in the intermolecular recognition process [15]. In the present study, docking was performed for the three ligands (MG-H1, AG, and DK) identified in the literature to confirm the interaction of these ligands at the RAGE binding site. The residues involved in MG-H1-RAGE interactions were LYS32, CYS79, ASN92, ALA3, SER91, SER2, LYS90, and ARG78 (Figure 3A). AG bound to LYS32 and CYS79 of RAGE (Figure 3B). The amino acid residues involved in DK-RAGE were ARG78, LYS90, ARG94, SER91, CYS79, GLN80, ALA3, and LYS32 (Figure 3C). The binding energy of the ligand-RAGE interaction from the lowest to the highest was DK-RAGE, MG-H1-RAGE, and AG-RAGE, with energies of −27.8524 kcal/mol, −25.7807 kcal/mol, and −15.5972 kcal/mol, respectively. Interestingly, DK had relatively stable binding energy levels with RAGE at values of –CDOCKER energy lower than that of MG-H1. This result indicated that DK was able to compete with MG-H1 for binding to the active site of RAGE.

To confirm the mechanism of MGO-induced renal damage in mesangial cells, MGO-mediated RAGE protein expression was determined using western blotting (Figure 3D). RAGE protein expression was increased approximately 8.4 times by MGO treatment compared to the normal group. (*** *p* < 0.001). However, DK pretreatment dramatically decreased the expression levels of RAGE in mesangial cells 24 h after MGO stimulation compared to the MGO-treated group (### *p* < 0.001) (Figure 3E). The MGO-induced upregulation of RAGE expression was reduced in mesangial cells pretreated with DK (20 μM) or AG (0.5 mM) by 84.36 ± 1.65% or 65.02 ± 0.87%, respectively, compared to the MGO group. To further confirm the effects of DK on the inhibition of intracellular AGEs accumulation, we performed immunofluorescence analysis using an AGEs antibody. AGEs were increased in mesangial cells cultured with 1 mM MGO, while pretreatment with DK (1, 5, and 20 μM) suppressed intracellular AGEs accumulation (Figure 3F).

These results demonstrate that DK has a competitive advantage over MGO in binding to the active site of RAGE, which may attenuate the AGE-RAGE interaction by suppressing the AGE-RAGE axis. Moreover, DK not only reduced the intracellular AGEs accumulation caused by MGO, but also decreased the downstream apoptosis cascade by suppressing the expression of RAGE protein in mesangial cells. Therefore, our results indicate that DK possesses strong potency to suppress AGE-mediated diabetic nephropathy.

### 3.4. Preventive Ability of DK against MGO-Induced Apoptotic Cell Death

**Figure 4:**
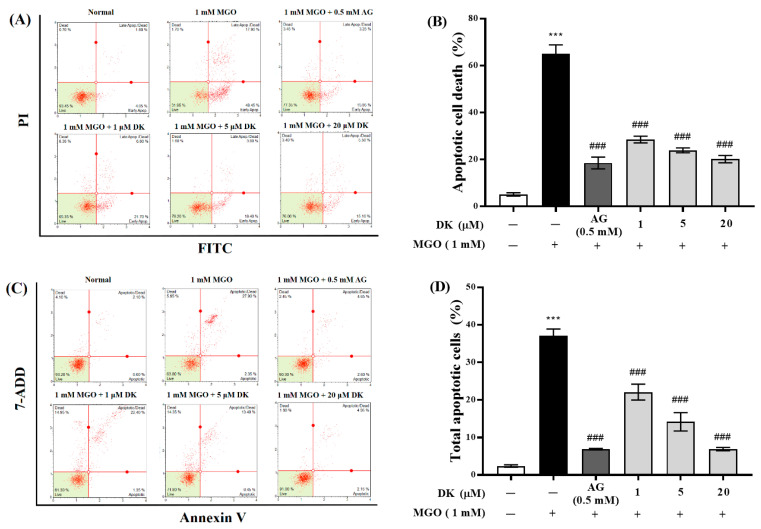

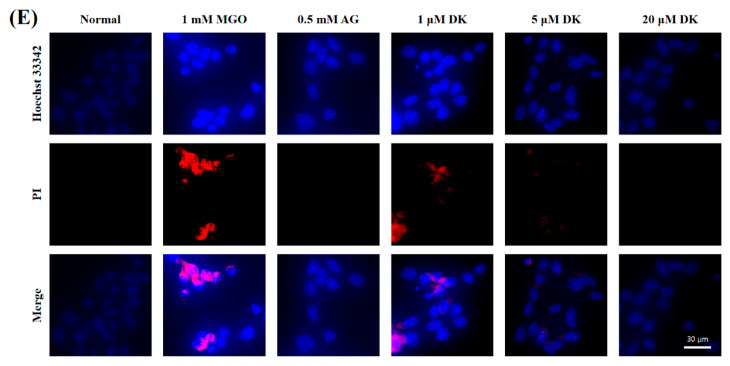
Antiapoptotic ability of DK on MGO-induced mouse glomerular mesangial cells. Cells were pretreated with DK (1, 5, and 20 μM) or AG (0.5 mM) for 1 h. Then, 1 mM MGO was added and incubated for 23 h. Total apoptotic cell death of mouse glomerular mesangial cells was determined using Annexin V & Dead Cell Assay Kit (Luminex, TX, USA). (**A**) The results of analyzing total apoptotic cell death using annexin V and PI double staining are shown in dot plots.; (**B**) The bar graph presents the percentage of total apoptotic cells (early apoptosis and late apoptosis); (**C**) Dot plots present the results of analyzing caspase 3/7; (**D**) The bar graph exhibits the percentage of caspase 3/7 dependent apoptotic cell death in mouse glomerular mesangial cells; (**E**) Representative images of Hoechst 33342/PI double staining. All data are expressed as mean ± standard deviation (*n* = 3) (*** *p* < 0.001 vs. nontreated cells and ### *p* < 0.001 vs. MGO-treated cells).

The induction of apoptosis after treating the cells with MGO was determined using the Muse ™ Annexin V & Dead Cell Kit and flow cytometry (Figure 4A). MGO treatment (1 mM) induced abnormal apoptosis in mesangial cells, and the number of apoptotic cells dramatically increased to 65.03 ± 3.80%, compared with that of the normal group (5.12 ± 0.70%) (*** *p* < 0.001). Furthermore, we found that DK pretreatment significantly reduced the number of apoptotic cells in a dose-dependent manner compared to the MGO-treated group (### *p* < 0.001). All test concentrations of DK (1, 5, and 20 μM) had a significant preventive effect on MGO-induced mesangial cell apoptosis, compared with the MGO-treated group (### *p* < 0.001) (Figure 4B). In particular, 20 μM of DK was the most effective at protecting mesangial cells from MGO-induced toxicity, and the total apoptotic cell ratio (20.17 ± 1.56%) was close to that of the 0.5 mM AG group (positive control, 18.5 ± 2.52%).

The apoptotic cascade of cells is known to induce protein substrate cleavage and apoptotic cell death. Quantitative measurement of apoptotic cells by casepase-3/7 activation was performed using flow cytometry with the Muse™ Caspase 3/7 assay kit (Figure 4C). Similar to the Annexin V assay, caspase-3/7 mediated apoptotic cell death was in-duced by MGO treatment, and the apoptotic cell ratio was significantly increased to 37.05 ± 1.84% compared to that of the normal group (2.38 ± 0.33%) (Figure 4D) (*** *p* < 0.001). However, all the test concentrations of DK (1, 5, and 20 μM) dramatically decreased caspase-mediated apoptosis in a dose-dependent manner, compared to the MGO-treated group (### *p* < 0.001). Particularly, it was confirmed that 20 μM DK pretreatment suppressed the MGO-induced apoptotic cascade in mesangial cells, thereby maintaining a level of live cells comparable to that seen in the control group. Moreover, the total apoptotic cell ratio (6.90 ± 0.43%) in the 20 μM DK pretreatment group was close to that in the AG group (6.87 ± 0.20%).

To further confirm the inhibitory effects of DK on MGO-induced apoptotic cell death, mesangial cells were double stained with Hoechst 33258/PI. It was confirmed that MGO-treated cells showed increased characteristics of a typical apoptotic body. However, DK pretreatment dramatically reduced the number of apoptotic cells (Figure 4E). These results suggested that DK is a potential inhibitor of MGO-induced apoptosis in renal cells.

### 3.5. Effect of DK on the Apoptosis-Related Protein Expression 

**Figure 5:**
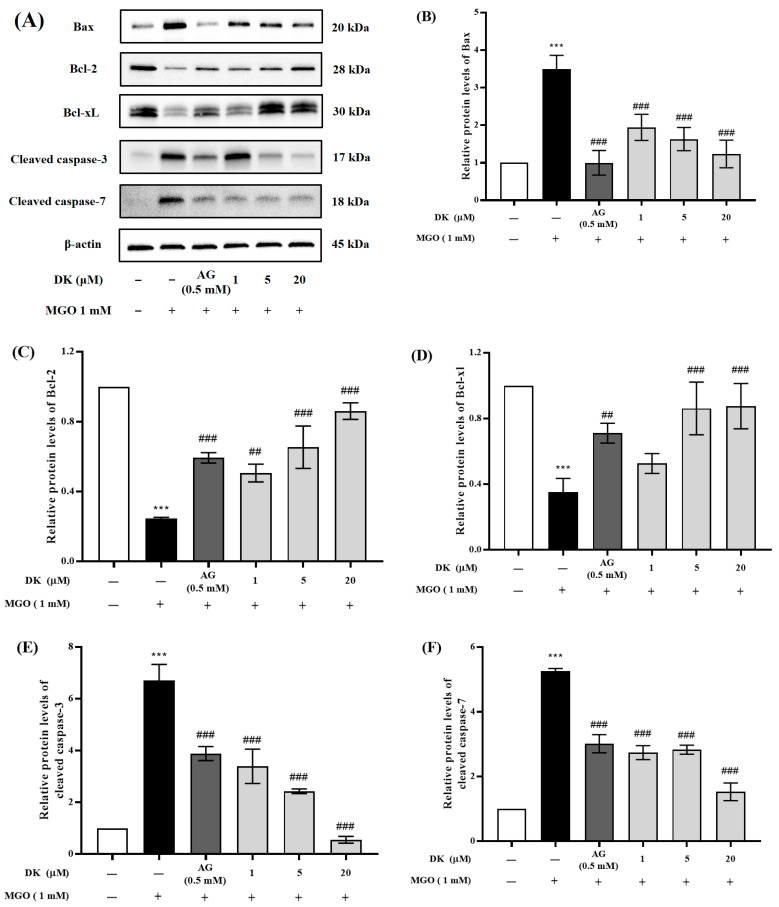
Preventive effect of DK on apoptosis-related protein expression in mouse glomerular mesangial cells. Cells were pretreated with DK (1, 5, and 20 μM) or AG (0.5 mM) for 1 h. Then, 1 mM MGO was added and incubated for 23 h. (**A**) The protein expression levels of antiapoptotic (Bcl-1 and Bcl-xL), proapoptotic (Bax), cleaved caspase-3, and cleaved caspase-7 were measured by Western blotting; (**B**–**F**) The bar graph demonstrates the relative protein expression band intensity level of Bax, Bcl-2, Bcl-xL, cleaved caspase-3, and cleaved caspase-7. All data are presented as mean ± standard deviation (*n* = 3) (*** *p* < 0.001 vs. nontreated cells and ## *p* < 0.01, ### *p* < 0.001 vs. MGO-treated cells).

Flow cytometric analysis revealed that MGO treatment induced apoptosis in mouse glomerular mesangial cells, whereas DK pretreatment inhibited MGO-induced renal cell apoptosis. To further confirm the mechanism of the protective effects of DK on MGO-induced renal cell apoptosis, the expression of antiapoptotic (Bcl-2 and Bcl-xL), pro-apoptotic (Bax), cleaved caspase-3, and cleaved caspase-7 proteins was measured (Figure 5A). As shown in Figure 5B–F, MGO treatment resulted in a significant increase in Bax, cleaved caspase-3, and cleaved caspase-7 protein expression and a significant de-crease in Bcl-2 and Bcl-xL protein expression in mesangial cells (*** *p* < 0.001). In contrast, pretreatment with DK (1, 5, and 20 μM) reduced the expression of Bax, cleaved caspase-3, and cleaved caspase-7 and significantly increased the antiapoptotic protein expression (Bcl-2 and Bcl-xL), thereby exerting the effect of DK on suppressing apoptotic cell death of MGO-induced mesangial cells (### *p* < 0.001).

### 3.6. Effect of DK on Nrf2/Glo-1/ARE Signaling Pathway 

**Figure 6:**
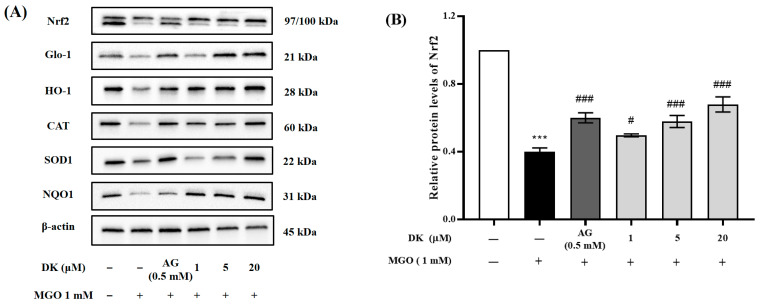

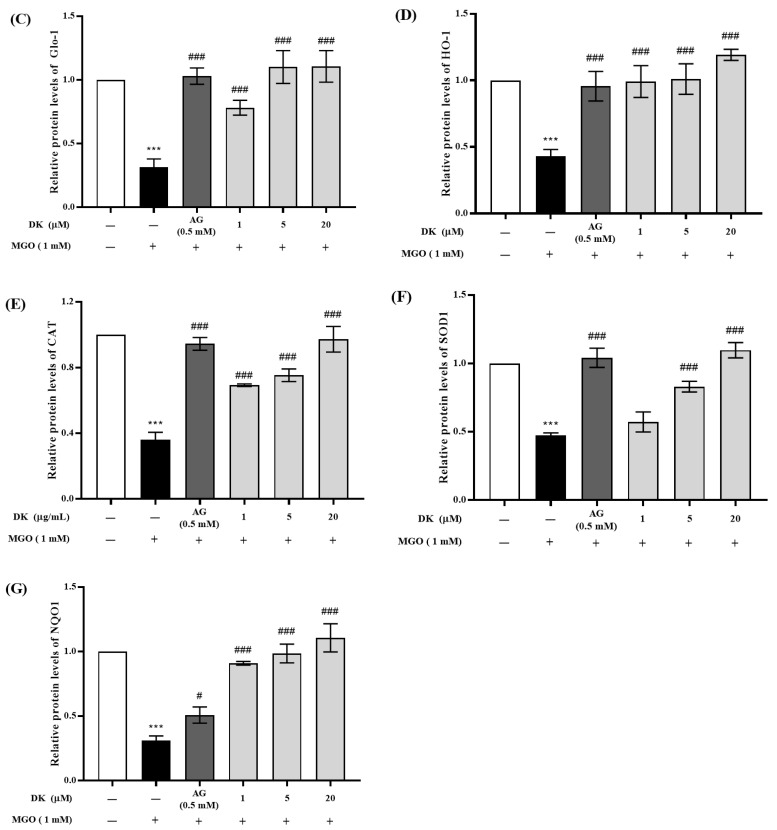
Effects of DK on Nrf2/Glo-1/ARE signaling pathway in mouse glomerular mesangial cells. Cells were pretreated with DK (1, 5, and 20 μM) or AG (0.5 mM) for 1 h. Then, 1 mM MGO was added and incubated for 23 h. (**A**) The protein expression levels of Nrf2 and Nrf2 downstream molecules, such as Glo-1, HO-1, CAT, SOD1, and NQO1, were measured by Western blot; (**B**–**G**) bar graph showing the relative protein expression band intensity level of Nrf2, Glo-1, HO-1, CAT, SOD1, and NQO1. The bar values are presented as mean ± standard deviation (*n* = 3) (*** *p* < 0.001 vs. nontreated cells and # *p* < 0.05, ### *p* < 0.001 vs. MGO-treated cells).

Because MGO-induced oxidative stress is one of the major contributors to diabetic complications, increasing the expression of ROS-related proteins, such as Nrf2/Glo-1/ARE, is a promising strategy for alleviating/preventing AGE-induced nephropathy. To further confirm the protective effect of DK under MGO-treated conditions, we investigated the protein expression levels of ROS-related signaling pathways, such as Nrf2, Glo-1, HO-1, NQO1, CAT, and SOD1 in mesangial cells by Western blotting. Figure 6A shows that treatment with MGO markedly decreased the expression of Nrf2 protein in mesangial cells, while preincubation for 1 h with DK (1, 5, and 20 μM) and then stimulation with MGO (1 mM) for 23 h significantly increased the expression of Nrf2 compared to that in the MGO-treated group (# *p* < 0.05, ### *p* < 0.001) (Figure 5B). MGO treatment dramatically downregulated Glo-1 expression in mesangial cells, whereas pretreatment with DK significantly upregulated the MGO-induced reduction in Glo-1 protein expression at a concentration of 1–20 μM (### *p* < 0.001) (Figure 5C). The activation of Nrf2-regulated genes by DK pretreatment was further confirmed through the alteration of downstream gene expression, such as HO-1, NQO1, CAT, and SOD1 (Figure 6D–G). MGO treatment dramatically decreased the protein expression levels of HO-1, NQO1, CAT, and SOD1, while DK (1, 5, and 20 μM) pretreatment significantly reversed this trend compared to the MGO-treated group (### *p* < 0.001). These results indicate that DK can suppress MGO-induced Nrf2, Glo-1, and ARE reduction, which may be part of its cytoprotective mechanism in mesangial cells.

### 3.7. Effect of DK on MAPKs’ Phosphorylation 

**Figure 7:**
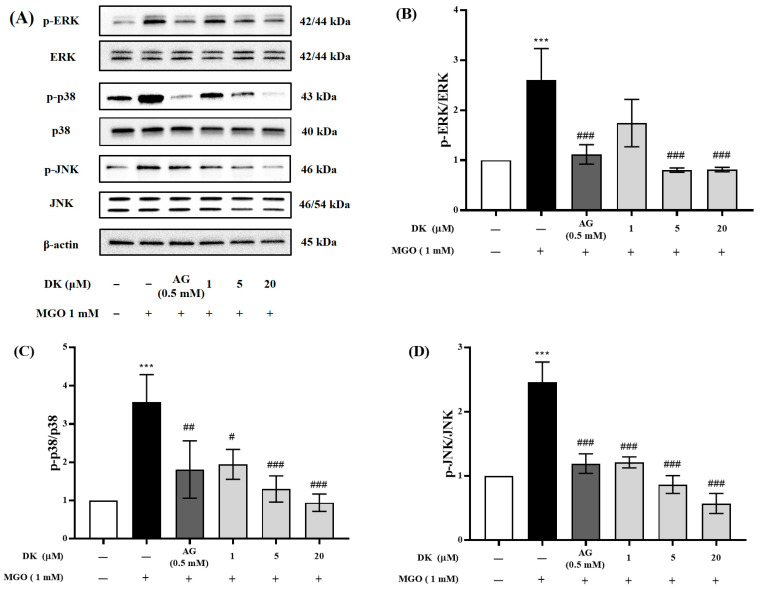
Effects of DK on MAPK phosphorylation in mouse glomerular mesangial cells. Cells were pretreated with DK (1, 5, and 20 μM) or AG (0.5 mM) for 1 h. Then, 1 mM MGO was added and incubated for 23 h. (**A**) The protein expression levels of p-ERK/ERK, p-p38/p38, and p-JNK/JNK were measured by Western blot; (**B**–**D**) bar values showing the relative protein expression band intensity level of p-ERK/ERK, p-p38/p38, and p-JNK/JNK. All data are presented as mean ± standard deviation (*n* = 3) (*** *p* < 0.001 vs. nontreated cells and # *p* < 0.05, ## *p* < 0.01, ### *p* < 0.001 vs. MGO-treated cells).

The phosphorylation-induced activation of MAPKs by MGO in mesangial cells is associated with an apoptotic cascade. Considering this, we further assessed the effect of DK on MAPK signaling phosphorylation, including ERK, p38, and JNK, during MGO-induced apoptosis using Western blotting (Figure 7A). As shown in Figure 7B–D, MGO treatment dramatically increased the levels of phosphorylated forms of ERK, p38, and JNK (*** *p* < 0.001). In contrast, pretreatment with DK (1, 5, and 20 μM) significantly decreased the phosphorylation of ERK, p38, and JNK in a dose-dependent manner (### *p* < 0.001).

## 4. Discussion

Advanced glycation end products (AGE) are yellow-brown compounds that are naturally formed by the glycation process, a nonenzymatic browning event in which the carbonyl groups of reducing sugars, such as glucose and fructose, as well as the amino group of proteins, form Schiff bases and Amadori products [3]. Furthermore, the heat treatment of food is an exogenous factor that generates AGE. Particularly, the production and accumulation of AGEs are promoted in diabetic patients who are continuously in a chronic hyperglycemic state, because blood glucose is the major source of carbonyl groups required for glycation reactions [16,17]. AGEs formed in the body have a strong affinity for long-lived proteins such as collagen. The formation of an irreversible bond by AGE-protein cross-linking not only accelerates the accumulation of AGEs in organs, but also directly causes aberrant protein structural modification. Furthermore, AGE-protein cross-linking induces diverse organ dysfunction through interfering with extracellular matrix–matrix and matrix–cell interactions in addition to AGE receptor-mediated mechanisms [18,19]. Many studies have revealed that the production and accumulation of AGEs in the basement membrane and endothelial and mesangial cells of the kidney are closely associated to the pathogenesis and progression of diabetic nephropathy (DN) [20]. Therefore, numerous researchers have postulated that inhibiting AGE formation, inhibiting AGE-collagen cross-links, and disrupting existing AGE-collagen cross-links are effective strategies for preventing AGE-associated DN. MGO is a dicarbonyl compound formed as a by-product of glycolysis and is one of the precursors required for AGE formation. Consequently, the MGO trapping reaction is regarded to be one of the mechanisms capable of effectively inhibiting AGE production. Several previous studies have demonstrated the antiglycation activity of terrestrial plant resources and bioactive compounds (phenolic and flavonoid compounds) derived from terrestrial plants [21,22]. Flavonoid compounds, in particular, are well known for their inhibitory effects on AGE generation via the MGO trapping reaction. Chlorogenic acid, quercitrin, and rutin isolated from *Houttuynia cordata* demonstrated a minimizing effect on AGE formation by creating mono- or di-MGO-conjugated adducts via the MGO trapping reaction [21]. Several studies have demonstrated that flavonoid compounds with specific structures, such as pyrocatechol, phloroglucinol, pyrogallol, and resorcinol, are more efficient than others at inhibiting AGE formation via MGO trapping [23,24,25]. Seaweed has traditionally been consumed, mostly in Asian coastal areas, and utilized as a traditional herbal medicine for a variety of ailments because it contains an abundance of physiologically active substances, such as polyphenols and polysaccharides. Recently, many scholars have been conducting research on the various physiological activities of seaweeds and their bioactive components. In particular, it has been demonstrated that polysaccharides (especially fucoidan), phlorotannins, and protein hydrolysates contained in seaweed can perform various biological functions, such as antidiabetic, anti-inflammatory, anticancer, antiviral, immunomodulatory, anticoagulant, antioxidant, antiobesity, and antiallergy [9,10]. Despite the abundance of bioactive substances in seaweed, little research has been conducted on its antiglycation activity. In a recent study, we revealed that seaweed has a strong antiglycation ability. Seaweed extracts exhibit antiglycation ability and brown algae have been proven to have a higher potential than red and green algae. Moreover, the antiglycation activity of seaweed was positively correlated with the content of phenolic and tannin substances among the bioactive components [26]. We reported that *Ecklonia cava* (edible brown algae; *E. cava*) extract, among seaweeds, is a rich source of bioactive substances that are effective in preventing DN caused by MGO-induced oxidative stress, as well as antiglycation reaction [27]. Because dieckol isolated from *E. cava* has a resorcinol structure, it is considered to have an inhibitory effect on AGE production through MGO trapping.

MGO, a highly reactive dicarbonyl intermediate metabolite among the precursors of AGE, has been linked to the development of diabetic microvascular complications, such as DN. The detoxifying processes of the body, such as the glyoxalase system, balance the AGEs produced in the body. Glo-1 overexpression not only suppresses intracellular MGO or MGO-derived AGE accumulation by improving the detoxifying ability in various cells, but it also promotes MGO conversion into nontoxic D-lactate [28]. Furthermore, stimulation of Glo-1 expression prevents the development of diabetic complications that are closely associated to microvascular complications by providing an enzymatic defense against MGO-mediated glycation. The accumulation of MGO and MGO-adducts induces toxicity in vascular endothelial cells and mesangial cells, triggering intracellular ROS production, apoptosis, and inflammation, ultimately leading to renal dysfunction [8,29]. MGO-induced RAGE protein expression triggers downstream signaling pathways, specifically promoting apoptosis via ROS-mediated MAPK phosphorylation, as well as apoptosis-related proteins such as Bax, Bcl, and cleaved caspase-3 [19,30]. Furthermore, when cells are subjected to oxidative stress caused by MGO, the interaction of the Nrf2-Keap1 complex in the cytoplasm is disrupted, and Nrf2 translocates to the nucleus, activating ARE-dependent genes such as HO-1, SOD, CAT, and NQO1. In the present study, MGO treatment not only reduced Nrf2 protein expression and the expression of Glo-1/ARE, a downstream signaling pathway, but also triggered an apoptotic cascade that caused renal cell damage [31,32].

The receptor for AGE (RAGE), a member of the immunoglobulin superfamily and an AGE-specific receptor, is closely implicated in diabetic complications caused by AGE. RAGE has been shown to be expressed in vascular cells, various types of epithelial cells, Müller cells in the retina, and almost all cell types in the kidney. A large body of evidence implies that DN is triggered by RAGE protein expression [3]. In particular, DN occurs in 35–45% of patients with diabetes and oxidative stress caused by AGE accumulation induces endothelial cell dysfunction, interstitial extracellular matrix deposition, and glomerular basement membrane thickening, resulting in glomerulosclerosis [19,33]. Moreover, various in vitro and in vivo studies have demonstrated that AGE–RAGE interaction stimulates intracellular ROS production and induces various signaling pathways, such as inflammation, fibrosis, and apoptosis [3]. Therefore, several researchers have attempted to elucidate the mechanisms involved in preventing or delaying the development of AGE-related DN. The inhibition of RAGE protein activation is considered the most important biomarker for the prevention of AGE-associated DN. Moreover, inhibition of AGE accumulation in renal cells and suppression of AGE–RAGE interactions are thought to be major mechanisms for DN prevention [19]. It was demonstrated that pretreatment of mesangial cells with *E. cava* extract not only suppressed MGO-induced AGE accumulation in renal cells, but also lowered RAGE protein expression [27]. In this study, molecular docking analysis demonstrated that the DK contained in *E. cava* had a comparative advantage over MG-H1 in the AGE–RAGE interaction and had competitive inhibitory potential. Moreover, DK pretreatment not only suppressed RAGE protein overexpression in mesangial cells caused by MGO treatment, but also protected kidney cells by stimulating the production of Glo-1, a detoxification enzyme. These results not only prove that DK can inhibit AGE-associated DN through various mechanisms, but also imply that DK is highly competitive in developing natural AGE inhibitors with low risk of side effects.

## 5. Conclusions

AGE is a complex and heterogeneous compound naturally produced by the glycation reaction of glucose and amino acids without the involvement of enzymes in the process of glucose metabolism. However, in the case of diabetic patients whose blood glucose concentration, a major component of AGE production, is continuously maintained at a high level, the production of AGE is accelerated and its accumulation in the body increases. Excessive generation of AGE beyond the level of detoxification in the body forms irreversible cross-links with long-lived proteins, such as collagen, and accumulates in the kidney, promoting the pathogenesis of AGE-associated DN. This study aimed to clarify the main mechanisms by which DK isolated from *E. cava* prevents AGE-related DN. Interestingly, molecular docking analysis demonstrated that DK has the potential to act as a competitive inhibitor of AGE–RAGE interaction, a key biomarker for the induction of AGE-mediated DN. Furthermore, DK was shown to protect renal cells from oxidative stress through the regulation of downstream signaling pathways, such as Nrf2/Glo-1/ARE, and apoptosis-related proteins regulated by the activation of RAGE expression in mouse glomerular mesangial cells. Consequently, we demonstrated that DK could prevent AGE-induced DN through various mechanisms and possesses potential as a therapeutic agent. However, this study evaluated the potential of DK for the prevention of diabetic nephropathy at the in vitro level using mouse glomerular mesangial cells, and it is considered necessary to verify it through in-depth research at the in vivo level.

## Data Availability

Data are contained within this article. Raw data are available from the corresponding author upon reasonable request.

## Supplementary Materials

The following are available online at https://www.mdpi.com/article/10.3390/antiox12030593/s1, Table S1: Antibody list.
