# Supplementary material for: Dieckol, Derived from the Edible Brown Algae Ecklonia cava, Attenuates Methylglyoxal-Associated Diabetic Nephropathy by Suppressing AGE–RAGE Interaction
Source: Antioxidants (Basel). 2023 Feb 27;12(3):593. doi: 10.3390/antiox12030593 (PMC10045168; doi:10.3390/antiox12030593)
Supplement: Supplementary file 1 [file antioxidants-12-00593-s001.zip › antioxidants-2242614-supplementary.pdf]

**Supplementary S1.**

Table S1. Antibody list

| Antibody | CAT NO. | Manufacturer | Size (kDa) |
|---|---|---|---|
| RAGE | MAB5328 | Millifore, Millerica, MA, USA | 48 |
| Glo-1 | Ab96032 | Abcam, Cambrige, MA, USA | 21 |
| Nrf2 | 12721s | Cell signaling, Danvers, MA, USA | 98 |
| HO-1 | 43966S | Cell signaling, Danvers, MA, USA | 28 |
| NQO1 | ab34173 | Abcam, Cambrige, MA, USA | 31 |
| CAT | 12980s | Cell signaling, Danvers, MA, USA | 60 |
| SOD1 | 2770s | Cell signaling, Danvers, MA, USA | 22 |
| Actin | sc-47778 | Santa Cruz biotechnology, Santa Cruz, CA, USA | 45 |
| p-ERK | 9101s | Cell signaling, Danvers, MA, USA | 42/44 |
| ERK | 9102s | Cell signaling, Danvers, MA, USA | 42/44 |
| p-JNK | 9251s | Cell signaling, Danvers, MA, USA | 46/54 |
| JNK | 9252s | Cell signaling, Danvers, MA, USA | 46/54 |
| p-p38 | 9211s | Cell signaling, Danvers, MA, USA | 43 |
| P38 | 9212s | Cell signaling, Danvers, MA, USA | 40 |
| Bax | 2774 | Cell signaling, Danvers, MA, USA | 20 |
| Bcl-2 | 3498 | Cell signaling, Danvers, MA, USA | 28 |
| Bcl-xL | 2762 | Cell signaling, Danvers, MA, USA | 30 |
| Cleaved caspase-3 | 9661 | Cell signaling, Danvers, MA, USA | 17/19 |
| Cleaved caspase-7 | 8438 | Cell signaling, Danvers, MA, USA | 18 |
